# From Visual Perception to Aesthetic Appeal: Brain Responses to Aesthetically Appealing Natural Landscape Movies

**DOI:** 10.3389/fnhum.2021.676032

**Published:** 2021-07-21

**Authors:** Ayse Ilkay Isik, Edward A. Vessel

**Affiliations:** Department of Neuroscience, Max Planck Institute for Empirical Aesthetics, Frankfurt am Main, Germany

**Keywords:** fMRI, neuroaesthetics, naturalistic stimuli, landscape movies, scene preference, aesthetic appeal, elemental affect, value

## Abstract

During aesthetically appealing visual experiences, visual content provides a basis for computation of affectively tinged representations of aesthetic value. How this happens in the brain is largely unexplored. Using engaging video clips of natural landscapes, we tested whether cortical regions that respond to perceptual aspects of an environment (e.g., spatial layout, object content and motion) were directly modulated by rated aesthetic appeal. Twenty-four participants watched a series of videos of natural landscapes while being scanned using functional magnetic resonance imaging (fMRI) and reported both continuous ratings of enjoyment (during the videos) and overall aesthetic judgments (after each video). Although landscape videos engaged a greater expanse of high-level visual cortex compared to that observed for images of landscapes, independently localized category-selective visual regions (e.g., scene-selective parahippocampal place area and motion-selective hMT+) were not significantly modulated by aesthetic appeal. Rather, a whole-brain analysis revealed modulations by aesthetic appeal in ventral (collateral sulcus) and lateral (middle occipital sulcus, posterior middle temporal gyrus) clusters that were adjacent to scene and motion selective regions. These findings suggest that aesthetic appeal *per se* is not represented in well-characterized feature- and category-selective regions of visual cortex. Rather, we propose that the observed activations reflect a local transformation from a feature-based visual representation to a representation of “elemental affect,” computed through information-processing mechanisms that detect deviations from an observer’s expectations. Furthermore, we found modulation by aesthetic appeal in subcortical reward structures but not in regions of the default-mode network (DMN) nor orbitofrontal cortex, and only weak evidence for associated changes in functional connectivity. In contrast to other visual aesthetic domains, aesthetically appealing interactions with natural landscapes may rely more heavily on comparisons between ongoing stimulation and well-formed representations of the natural world, and less on top-down processes for resolving ambiguities or assessing self-relevance.

## Introduction

Interactions with the natural environment can be highly impactful and aesthetically rewarding. Natural landscapes are rich sources of beauty, pleasure, awe and fascination (Kaplan, 1995; Keltner and Haidt, 2003; Eisenberger et al., 2010; Augustin et al., 2012; Brielmann et al., 2020), aesthetic properties that likely contribute to the documented beneficial effects of spending time in nature, such as improved cognition, creative problem solving, life satisfaction, mental health and well-being (Atchley et al., 2012; Bratman et al., 2015a, b; Twohig-Bennett and Jones, 2018; Chang et al., 2020).

Whereas a wealth of detail is known about how the human brain represents what is in our visual environment (e.g., category-specific representations of content; Malach et al., 1995; Kanwisher et al., 1997; Epstein and Kanwisher, 1998; Haxby et al., 2001), how that environment is changing (Tootell et al., 1995) and how we interact with it (Ungerleider and Mishkin, 1982; Goodale and Milner, 1992; Kravitz et al., 2011b) there is much less known about how such interactions become endowed with aesthetic qualities. It is clear that aesthetic evaluations involve not only the visual system but also processes for making meaning and for computing valuation (see Chatterjee and Vartanian, 2014; Vessel, 2020). Yet there is little agreement on precisely how neural representations of visual content inform computations of aesthetic appeal.

More specifically, within visual cortex it is unclear whether information about the aesthetic appeal of a scene is present in the same cortical regions that represent perceptual aspects of that scene such as content, layout or movement. The most well studied scene-selective region using functional magnetic resonance imaging (fMRI) is in the parahippocampal gyrus (PHG) or adjacent collateral sulcus (CoS); when identified by a contrast of activation for scenes vs. images of isolated objects, this region is referred to as the “parahippocampal place area” (PPA; Epstein and Kanwisher, 1998). It is thought to represent aspects of a scene that can be used to identify it as a particular place (Epstein, 2020) including its spatial boundaries (Park et al., 2011), 3D structure and geometry (Epstein and Ward, 2010; Walther et al., 2011), and relevant contextual associations (Bar and Aminoff, 2003). Two additional scene-selective regions have also been identified, namely the retrosplenial cortex (RSC; Aguirre et al., 1996; Epstein, 2008) on the medial surface and the “occipital place area” (OPA; Nakamura et al., 2000; Dilks et al., 2013) on the lateral surface.

Several studies have reported increased activity in or near the PPA in response to preferred scenes, but the evidence is far from conclusive. Yue et al. (2007) reported greater activity for preferred vs. non-preferred scenes (a mixture of indoor and outdoor) in right, but not left PPA. A study of ‘sublime’ natural landscapes found increased activity in an extensive portion of the ventral occipitotemporal cortex (VOT) stretching from fusiform gyrus to PHG and underlying posterior hippocampus (Ishizu and Zeki, 2014); yet as no scene-selective localizer was performed, the location of activation relative to PPA was unclear. Another study reported that attractiveness ratings of natural landscapes were correlated with subthreshold activity changes in PHG and CoS, but that only activity in the object-selective lateral occipital complex (LO), and not place-selective PPA, was significantly correlated with place attractiveness (Pegors et al., 2015). Ratings of beauty of interior architecture images have also been found to correlate with activity in PHG and middle occipital gyrus (MOG; Vartanian et al., 2013b). Finally, a study comparing several aesthetic domains found spatial patterns of activity in VOT that were predictive of aesthetic appeal for natural landscapes, and stronger responses in both PPA and object-selective ventral object area (VOA) for appealing vs. non-appealing natural landscapes, interior and exterior architecture (Vessel et al., 2019).

Though less directly relevant for scenes, studies investigating whether aesthetic appeal of *faces* is represented in regions of the face-selective network have reported mixed results, with some studies showing effects in fusiform face area (FFA; Kanwisher et al., 1997) (Chatterjee et al., 2009; Pegors et al., 2015) and others not (Vartanian et al., 2013a; Hartung et al., 2019).

Our primary aim was thus to identify modulations correlated with aesthetic appeal in visual brain regions for experiences with landscapes, and to understand how such activity relates to well-characterized representations of perceptual features such as scene layout. To do so, we curated a set of artistically engaging video clips of natural landscapes (30 s duration). Natural landscapes have a strong capacity to invoke aesthetic engagement (Kant, 1790/1987; Brielmann et al., 2020). Importantly, an analysis of scene shape is critical for evaluation of landscape videos, which should lead to strong engagement of scene-selective regions (PPA, OPA, and RSA). We identified these regions using independent functional localizers in each individual and used them to directly test whether aesthetic appeal modulated activity in feature-selective regions of visual cortex.

The use of videos of natural scenes adds another feature, motion, whose representation in visual cortex is also well characterized. Similar to the scene-selective areas, it is unclear whether area hMT+ (human middle temporal complex), a core region for the computation of visual motion (Tootell et al., 1995; Beauchamp et al., 2003), is modulated by aesthetic appeal. To date only one study, using kinetic dot patterns, has tested whether activity in hMT+ was greater for aesthetically appealing patterns of motion (Zeki and Stutters, 2012). Although they did not use a functional localizer approach, group-level sensitivity for aesthetic appeal did appear to overlap with motion selectivity in the approximate location of hMT+. We therefore included an independent localizer for motion selectivity and tested whether hMT+ activity was modulated by aesthetic appeal.

In addition to this primary aim, our design also allowed us to address two further questions. Our secondary aim sought to test whether aesthetically appealing experiences with natural landscapes engage medial prefrontal cortex (*m*PFC), orbitofrontal cortex (OFC) or nodes of the default-mode network (DMN). Many studies have reported that aesthetically appealing stimuli are associated with greater fMRI activation in portions of prefrontal cortex that support valuation and emotion (Brown et al., 2011; Chatterjee and Vartanian, 2016). This finding appears particularly robust for cultural artifacts such as artwork. Modulations of aesthetic appeal have been reported below the superior rostral sulcus (SRS) in medial orbitofrontal cortex (*m*OFC) for visual artwork (Kawabata and Zeki, 2004; Lacey et al., 2011) and music (Blood and Zatorre, 2001). Sensitivity to aesthetic appeal has also been reported in or above the SRS in *m*PFC for visual artworks (Vartanian and Goel, 2004; Vessel et al., 2012), abstract patterns (Jacobsen et al., 2006), architecture (Vartanian et al., 2013b) and for non-visual stimuli such as music (Ishizu and Zeki, 2011) and mathematical beauty (Zeki et al., 2014). In addition, several nodes of the DMN, a network of functionally connected regions that mediates aspects of internally directed thought (Raichle et al., 2001; Andrews-Hanna, 2012) have also been found to be engaged for artworks rated as strongly moving (Vessel et al., 2012, 2013; Belfi et al., 2019).

However, the question of whether portions of the prefrontal cortex or DMN are engaged by aesthetically appealing natural scenes is an unsettled issue. Using a whole-brain activation analysis, one study reported effects in prefrontal regions for aesthetic appeal of diverse scenes (Kirk, 2008) while another reported no effect (Yue et al., 2007). A study using images of natural landscapes reported activity correlated with aesthetic appeal in *m*PFC, but only when small-volume correction was applied using an *a priori* defined region-of-interest (ROI; Pegors et al., 2015), whereas a recent study using awe-inducing videos of natural landscapes reported less activation in *m*PFC and other DMN nodes (posterior cingulate cortex; PCC) when compared to neutral videos (VanElk et al., 2019). Using multivariate methods, three separate studies have found that patterns of activation in portions of *m*OFC, *m*PFC and the DMN contain information about aesthetic appeal of either natural landscapes (Pegors et al., 2015; Vessel et al., 2019) or about rated valence of affective pictures (Chikazoe et al., 2014); however, one of these found no differences in average activation for high vs. low appeal natural landscapes, despite strong pattern-related predictability (Vessel et al., 2019). In contrast, the evidence for increased *m*OFC/*m*PFC activation for attractive faces is more consistent (Aharon et al., 2001; O’Doherty et al., 2003; Kim et al., 2007; Smith et al., 2010; Tsukiura and Cabeza, 2011). We therefore sought to assess whether aesthetically appealing movies of natural landscapes would lead to significant modulation of average fMRI activity in *a priori* defined nodes of the DMN (including *m*PFC) or orbitofrontal cortex.

Our third aim was to assess whether feature-selective visual regions show increased functional connectivity with reward or DMN regions for aesthetically appealing experiences with natural landscapes. One possible mechanism by which visual information is transformed into a representation of aesthetic appeal is through an increase in coordinated activity between visual regions and subcortical reward, prefrontal or DMN regions. An increase in functional connectivity between content-related visual regions and ventromedial prefrontal cortex (vMPFC) has been reported during valuation judgments of t-shirt designs (Lim et al., 2013), and also between auditory cortices and nucleus accumbens for rewarding music (Salimpoor et al., 2013). Due to their dynamically changing nature, videos are more likely to induce fluctuations in visual regions that can then be tracked across the brain. We computed a measure of functional connectivity between sensory, reward and DMN regions for each video, and tested whether this connectivity was correlated with ratings of aesthetic appeal.

In addition to increasing engagement (Cutting et al., 2011; van der Meer et al., 2020) and allowing for a more robust measure of functional connectivity, the use of videos has several other advantages over images. Narrative film can create attentional synchrony across participants (Loschky et al., 2015). Yet natural landscape videos also appear to reduce agreement across individuals for which clips they find most appealing, compared to static images of landscape (Isik and Vessel, 2019). Methodologically, this allows for better separation of fMRI effects attributable to differences in content vs. differences in appeal: when people don’t find the same stimuli appealing, stimulus features can be ruled out as the primary cause of appeal-related modulations of brain activity (Vessel et al., 2012). Movies of landscapes also better reflect the ways people typically engage with natural environments, such as when one walks through a forest to explore, changes their view to take in an expansive vista or watches light and clouds move across a landscape. The popularity of artistic drone and time-lapse footage of natural landscapes on video sharing and social media websites attest to the power of natural landscape to engage and move us.

To test these hypotheses, twenty-four observers watched 30 s video clips of natural landscapes while being scanned using fMRI. During the clips, observers were asked to continuously rate their enjoyment using a hand-held dial. After the clip ended, observers then used the same dial to rate the overall intensity of their aesthetic experience. We found that ROIs sensitive to specific perceptual features such as scene layout and motion were strongly activated by the landscape movies, but were not clearly modulated by overall aesthetic appeal. Rather, a whole-brain analysis revealed several regions sensitive to aesthetic appeal of dynamic natural landscapes that were adjacent to, or only partially overlapping with, these ROIs representing specific perceptual features. Beyond the visual system, we observed that subcortical regions of the basal ganglia were modulated by aesthetic appeal, but that no modulation by aesthetic appeal could be detected in prefrontal and DMN regions using reasonable thresholds. Finally, functional connectivity between visual ROIs and reward or DMN ROIs was only weakly modulated by aesthetic appeal, failing to survive a stringent corrections threshold.

These results suggest that features that drive aesthetic appeal *per se* are not represented in core visual regions that are selective for specific visual features such as scene layout and motion, but rather in cortex adjacent to these regions. Additionally, we present evidence that, at least for movies of natural landscapes, the neural basis for aesthetic appeal does not depend on either large-scale activation in prefrontal cortex nor on functional connectivity between content-selective visual regions and reward or DMN regions.

## Materials and Methods

### Participants

Twenty-six participants were recruited for this study and paid for their participation. Two participants were excluded, one due to excessive motion (mean framewise displacement > 0.5 mm) and one due to missing behavioral data, leaving a final group of 24 participants (13 f; 18 right-handed, 24.7 ± 6.8 years of age). Informed consent was obtained in accordance with a protocol approved by the Medical Ethics Committee of Goethe University Frankfurt and was signed by all participants before the experiments.

### Stimuli and Procedures

Stimuli were 30 s video clips of landscapes collected from video streaming websites (e.g., YouTube or Vimeo) or from non-narrative cinematic films. The movies consisted of aerial drone shots, point-of-view shots or time-lapse photography depicting different types of natural landscapes (e.g., mountain, forest, ocean, river). They had clear artistic intent conveyed through the selection of depicted content, light conditions, use of camera techniques, and presence of changes throughout the duration of the clip (e.g., movement of camera, changes in weather or time of day). To ensure that aesthetic engagement was mainly driven by the landscape content, videos did not include human beings, animals or other objects. Forty movies were clipped to 30 s length using Adobe Premier Pro and saved with the same aspect ratio (16:9), resolution (1280 × 720 px) and video compression method (H.264). Movie stimuli were presented using PsychoPy2 (v1.85.2) and MovieStim3 (Peirce, 2008) at the center of the screen (approximate field-of-view 27°× 15°). All experimental stimuli were delivered using MRI-compatible VisuaStim goggles system (Resonance Technology) with a display resolution of 800 × 600, 30° horizontal field of view and a refresh rate of 60 Hz.

**FIGURE 1:**
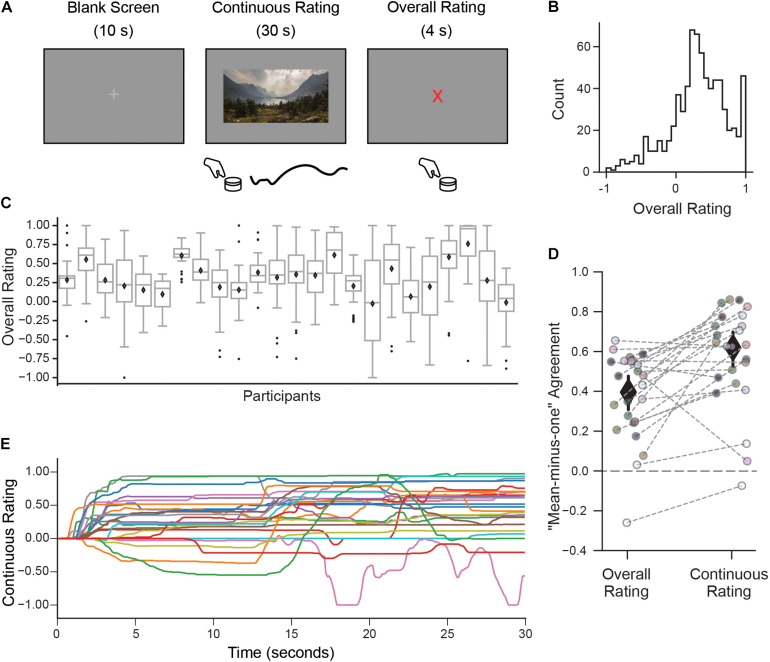
A continuous rating paradigm for assessing individual aesthetic enjoyment of landscape videos. **(A)** Schematic description of one experimental trial measuring continuous aesthetic responses to dynamically changing visual experiences. Participants viewed 30 s movie clips of landscapes while making continuous ratings of their moment-to-moment enjoyment. This was followed by an overall rating indicating the intensity of the aesthetic experience from the whole movie. Both responses were given using a rotary dial. **(B)** The histogram of overall ratings collapsed across all participants shows that most ratings were above the ‘indifference’ point (marked here as 0). **(C)** However, the distribution of overall ratings as illustrated with boxplots suggests marked differences across people in terms of their overall aesthetic ratings (Diamond is the mean, center line is the median, bottom and top edges of the box indicate the 25th and 75th percentiles. Whiskers extend to the most extreme data points and black circles indicate individual outliers). **(D)** Measure of agreement across participants (“mean-minus-one” correlation, see Materials and Methods) for overall and continuous ratings. Each circle represents the MM1 value for one participant and the gray dashed lines connect the MM1 values computed with overall and continuous ratings for the same participant. Diamonds are mean MM1 values across participants; error bars are 95% confidence intervals. **(E)** Continuous rating traces given by each participant for one movie clip (with the median MM1c value). In general, movie clips were rated differently by different participants, in terms of their overall mean liking and variability over time.

Participants completed a 6-min resting state scan in which they were asked to fixate a central fixation cross on a gray background. In the following four runs (8 movies each), participants viewed 32 landscape movies in an order counterbalanced across participants. Each movie trial began with a 10 s fixation period, followed by a 30 s movie presentation with no fixation requirement, followed by a 4 s response period (Figure 1A). A fifth run contained repeated presentations of 8 movies shown during the earlier runs (same movies for all participants) as a way to assess consistency of neural (not reported here) and behavioral responses. One movie clip was removed from further analysis due to a technical problem, leaving 31 trials in the main analysis and 7 trials in the consistency analysis. The duration for each run was 6 min and 4 s including a 2 s initial and 10 s final blank period.

During the videos, participants were asked to make continuous evaluations of their aesthetic enjoyment using a rotary dial. They were told to move the dial to the right if they were feeling more enjoyment or pleasure and to the left if they were feeling less enjoyment or pleasure when watching the video clip (“*How much are you enjoying the clip at each moment?”*). After the video clip finished playing, participants were given 4 s and asked to make a summary aesthetic judgment (“*How intense was your aesthetic experience overall?”*). Participants were instructed to give ratings of their subjective aesthetic experience of the clips and that they should base their judgments on their personal experience. They were told that they might have a more intense aesthetic experience for many different reasons, such as a clip being experienced as beautiful, profound or emotionally moving. Responses were collected using a custom-made MRI compatible rotary dial (Current Designs, Philadelphia, United States) positioned at their side. The dial had stops at the 9 and 3 o’clock positions and haptic feedback on the 12 o’clock position indicating extreme negative, extreme positive and neutral enjoyment, respectively. Participants were instructed to position the dial at the 12 o’clock neutral position before the start of each trial. This design allowed the participants to give ratings ranging from negative to positive without the need for visual feedback that would interfere with a visual experience. Before the experiment, observers were given practice with visual feedback on how to use the dial to help them calibrate their responses.

After the video runs, participants completed two functional localizer scans: a place, face, object, body localizer (PFOB localizer) and motion (hMT+) localizer. The PFOB localizer contained blocks of places, faces, objects, scrambled objects and bodies without faces. This scan consisted of five blocks of each stimulus type, during which the participant performed a “1-back” task responding each time an exact repeat of an image appeared. Each block contained 16 stimulus images (image size = 20°× 20°) each presented for 800 ms with a 200 ms inter-stimulus-interval. In the hMT+ localizer participants viewed alternating blocks of biological point-light actions, position-scrambled point-light controls, and static frames of the scrambled point-light control condition (Grossman and Blake, 2002; Vangeneugden et al., 2014). Each pattern consisted of 12 dots; the scrambled animations contained the same motion vectors as the biological ones, but the initial starting positions of the dots were randomized (details of the biological motion sequences are described in Grossman and Blake, 2002). There were 14 stimulation blocks for each condition and four rest blocks. The stimulation blocks consisted of five dot-pattern stimuli presented for 1 s with a 1 s inter-stimulus-interval. The dot stimuli appeared in a rectangle that subtended approximately 20°× 22° visual angle. Again, participants performed a “1-back” task to maintain attention. For both functional localizer tasks participants were instructed to maintain strict fixation. Stimuli were displayed using Matlab (Mathworks, Inc., 2016b) with Psychophysics Toolbox (Brainard, 1997, Pelli, 1997, Psychtoolbox-3).

### Data Acquisition and Preprocessing

#### Behavioral Data

Data for both continuous and overall ratings were scaled to range between −1 and 1. Continuous data were collected with a sampling rate of 60 Hz and down sampled to 10 Hz. Overall ratings given at the end of each movie were categorized into four bins and were used in the whole-brain activation analysis and ROI activation analysis. The binning was done for each participant separately by discretizing the values of the overall ratings into four bins based on quartiles.

#### fMRI Data Acquisition

Functional magnetic resonance imaging data collection was carried out at University of Frankfurt’s Brain Imaging Center, using a 3-T Siemens Trio scanner and an eight-channel phased array head coil (Siemens). The blood oxygen level dependent (BOLD) signal was measured using 32 3 mm slices (2 mm + 50% distance factor) that were acquired in an oblique orientation of approximately 20° to the anterior commissure-posterior commissure (AC-PC) axis to reduce the signal dropouts in the ventral prefrontal cortex (in plane resolution 3 mm × 3 mm, TR = 2s, TE = 30 ms, Flip Angle = 90°). The standard Siemens sequence was customized to include an additional slice-wise z shimming (−1.3 mT/m). Before every functional scan a short EPI (3 TR) with opposite phase encoding direction was collected for use in phase unwarping during preprocessing.

A high-resolution (1 mm^3^) anatomical volume (MPRAGE sequence) was obtained after the functional scans. Data were converted from DICOM to NIFTI and structured according to the BIDS standard (Gorgolewski et al., 2016) using Heudiconv (version 0.5^1^). Neuroimaging data was preprocessed using fMRIPprep 1.1.8 (Esteban et al., 2018). The details of the anatomical preprocessing steps for the T1 weighted (T1w) image can be found here: https://fmriprep.org/en/1.1.8/workflows.html#. As for the functional scans, for each of the 8 BOLD runs per participant, first a reference volume and its skull-stripped version were generated using a custom methodology of fMRIPrep. A deformation field to correct for susceptibility distortions was estimated based on two echo-planar imaging (EPI) references with opposing phase-encoding directions using 3dQwarp (AFNI) (Cox and Hyde, 1997)^2^. Based on the estimated susceptibility distortion, an unwarped BOLD reference was calculated for a more accurate co-registration with the anatomical reference. The BOLD reference was then co-registered to the T1w reference using bbregister (FreeSurfer) which implements boundary-based registration (Greve and Fischl, 2009). Co-registration was configured with nine degrees of freedom to account for distortions remaining in the BOLD reference. Head-motion parameters with respect to the BOLD reference (transformation matrices, and six corresponding rotation and translation parameters) were estimated before spatiotemporal filtering using MCFLIRT [FSL 5.0.9 (Jenkinson et al., 2012)]. The BOLD time-series were resampled into their original, native space by applying a single, composite transform to correct for head-motion and susceptibility distortions. Automatic removal of motion artifacts using independent component analysis (ICA-AROMA, Pruim et al., 2015) was performed on the preprocessed BOLD time-series in MNI space after spatial smoothing with an isotropic, Gaussian kernel of 6 mm FWHM (full-width half-maximum). Corresponding “non-aggressively” denoised runs were produced after such smoothing. Several confounding time-series were calculated based on the preprocessed BOLD: framewise displacement (FD), DVARS (Derivative of rms VARiance over voxelS) and three region-wise global signals. FD and DVARS were calculated for each functional run, both using their implementations in Nipype (Power et al., 2014). The three global signals were extracted from the cerebrospinal fluid (CSF), the white matter (WM), and the whole-brain masks. Additionally, a set of physiological regressors were extracted to allow for component-based noise correction [CompCor (Behzadi et al., 2007)]. Principal components were estimated after high-pass filtering the preprocessed BOLD time-series (using a discrete cosine filter with 128s cut-off) for the anatomical correction (aCompCor) and temporal (tCompCor) variants. Six tCompCor components were then calculated from the top 5% variable voxels within a mask covering the subcortical regions. This subcortical mask was obtained by heavily eroding the brain mask, which ensures it does not include cortical GM regions. For aCompCor, six components were calculated within the intersection of the aforementioned mask and the union of CSF and WM masks calculated in T1w space, after their projection to the native space of each functional run (using the inverse BOLD-to-T1w transformation). Gridded (volumetric) resamplings were performed using antsApplyTransforms (ANTs), configured with Lanczos interpolation to minimize the smoothing effects of other kernels (Lanczos, 1964). The internal operations of fMRIPrep use Nilearn 0.4.2 (Abraham et al., 2014) mostly within the functional processing workflow.

### Data Analysis

#### Agreement Analysis

Agreement for overall and continuous ratings across participants was quantified by using a “mean-minus-one” (MM1) correlation measure (Vessel et al., 2018). To calculate the MM1 scores for overall ratings we took each individual’s ratings and computed Pearson correlations with the average ratings of all other (N-1) individuals. This procedure produces an *r* score for each individual indicating how much this person is in agreement with the rest of the participants. We applied a similar leave-one-out framework to calculate a measure of agreement for continuous ratings in which one participant’s rating timecourse was correlated with the average of all others (mean-minus-one “continuous” correlation, MM1c). This procedure results in a participant-by-movie array that was then averaged across movies to obtain one value per participant indicating how much this person was in agreement with the rest of the participants for their moment-to-moment ratings. To obtain average across-observer MM1c scores, we first transformed individual *r*-values to *z*-values, computed the mean and 95% confidence intervals, and then transformed those scores back to *r*-values (Bronstad and Russell, 2007).

To quantify idiosyncratic and shared contributions to the overall aesthetic ratings we estimated proportions of “shared” and “private” taste with a variance decomposition method (Germine et al., 2015) with a modification proposed by Martinez et al. (2020) to more correctly handle participants who show negative agreement. By using the movie overall ratings from the last run of the experiment which contained repetitions of previously seen movies (nr of movies = 7) we partitioned the total variance of responses into non-repeatable vs. repeatable variance and then subdivided the repeatable variance into shared vs. individual variance.

#### Motion Energy Calculation

A measure of motion energy for each video clip was computed by applying a Gabor jet simple cell model (Yue et al., 2012; Margalit et al., 2016) to each frame and then computing a vector of framewise differences in the model output. This model was used as opposed to a simpler pixelwise motion energy metric because it more closely resembles the information thought to be encoded by the early visual system (Lades et al., 1993). To explore whether participants’ aesthetic ratings were affected by the motion energy in the movies we calculated Pearson correlations between participants’ continuous rating scores and the framewise motion energy values (downsampled to 10 Hz to match the sampling rate of the ratings) as well as participants’ overall ratings and the average motion energy value for each movie.

#### Identification of Participant-Specific Regions of Interest

**FIGURE 2:**
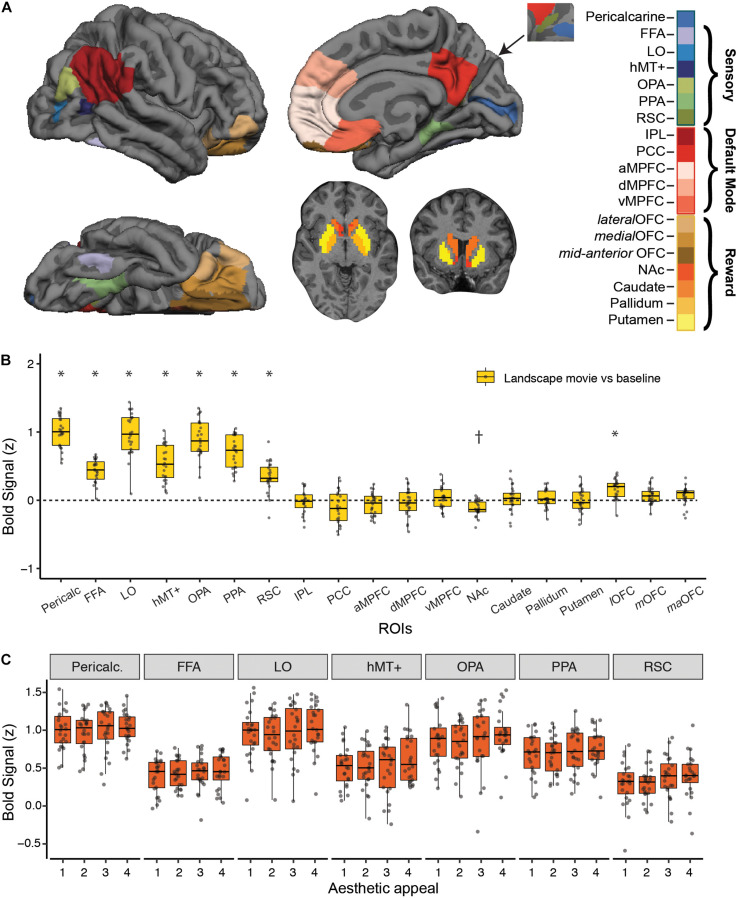
Strength of BOLD activation vs. baseline and effect of aesthetic appeal in visual, default-mode and reward networks. **(A)** Nineteen *a priori* regions of interest (ROIs) from three networks were identified and mean signal from each ROI was extracted for each participant. **(B)** Activations in each ROI for viewing landscape movies (vs. baseline). All visual ROIs were strongly engaged by landscape movies, with pericalcarine, LO, and OPA showing the strongest activation. *Indicates greater and ^†^indicates lesser activation compared to a resting baseline **(C)** Beta values from a univariate activation analysis in *a priori* visual ROIs did not reveal significant differences across 4 different levels of aesthetic appeal. In **B**, **C**, boxplots display the median (center line), the 25th and 75th percentiles (box edges) and extrema (whiskers). Filled circles represent scores for each participant and points outside the reach of the whiskers are individual outliers; *N* = 24.

Participant-specific maps of the DMN were obtained using the rest scan. High-pass filtering at 0.005 Hz and spatial smoothing with 6-mm FWHM Gaussian filter were applied using FSL to the non-aggressively cleaned fmriprep output in MNI space (Pruim et al., 2015). Then, we applied group-average independent component analysis (ICA) using FSL’s MELODIC tool. Because we were interested in obtaining macroscale functional networks we selected a lower order model with 20 components (Ray et al., 2013). The spatial maps from the group-average analysis were used to generate participant-specific versions of each map and associated timeseries using dual regression (Beckmann et al., 2009). First, for each participant, the group-average set of spatial maps was regressed (as spatial regressors in a multiple regression) into the participant’s 4D space-time dataset. This results in a set of participant-specific timeseries, one per group-level spatial map. Next, those timeseries were regressed (as temporal regressors, again in a multiple regression) into the same 4D dataset, resulting in a set of participant-specific spatial maps, one per group-level spatial map. These ICA components were compared to a set of predefined network maps (Smith et al., 2009) using Pearson correlation. The component with the highest correlation to the Smith et al. DMN map (Smith et al., 2009) was then thresholded (FDR < 0.05, cluster size > 100 voxels) and visually inspected to ensure that its spatial distribution appeared similar to the canonical DMN. A set of five subregions (anterior medial prefrontal cortex, aMPFC; dorsal medial prefrontal cortex, dMPFC; ventral medial prefrontal cortex, vMPFC; posterior cingulate cortex, PCC; inferior parietal lobule, IPL) were identified in each hemisphere by masking the thresholded DMN maps with a set of “master” ROIs delineated on the Freesurfer fsaverage brain and transformed back to MNI space (Belfi et al., 2019). These master ROIs, which each covered a contiguous region of cortex larger than the corresponding DMN subregion in any one participant, were drawn from the distribution of locations of these subregions observed in an independent sample of 16 participants (Vessel et al., 2019). This method was used in order to identify previously characterized, spatially specific nodes of the DMN from each individual’s own functional connectivity in a manner that required minimal manual intervention. These maps were then transformed back to participants’ T1 space for further analysis (for a surface visualization of the master ROIs see, Figure 2A).

Data from the PFOB and hMT+ localizers were analyzed with standard random-effects general linear models (FSL FEAT) after high pass filtering with 100 s cut-off. For the PFOB localizer we used separate predictors for face, object, scene, body and scrambled object blocks. Face-selective voxels were identified by contrasting face blocks to the average of the object, scene, and scrambled object blocks and object-selective voxels were identified by contrasting object blocks to the average of the face, scene and scrambled object blocks. Similarly, scene-selective voxels were identified by contrasting scene blocks to the average of the face, object and scrambled object blocks. For the hMT+ localizer we modeled separate predictors for position scrambled point-light controls, static frames of the scrambled point-light control conditions and biological point-light actions. To localize hMT+, position-scrambled point-light control stimuli (consisting of moving dots with local, non-coherent motion) were contrasted with static frames of these scrambled actions (Vangeneugden et al., 2014). The biological point light action stimuli, typically used to localize pSTS, were not used for this study.

To define participant-specific ROIs we adopted the group-constrained participant-specific ROI definition procedure (Fedorenko et al., 2010; Julian et al., 2012). This procedure involves taking individual participants’ thresholded (*z* > 2.3, cluster threshold *p* < 0.05) activation maps and overlaying them on top of each other in a common stereotaxic space (MNI) to create probabilistic overlap maps for each contrast of interest. We created these probabilistic maps for each contrast and transformed them to Freesurfer’s fsaverage surface (mri_vol2surf) to be able to visualize and delineate each target region of interest. The parahippocampal place area (PPA), occipital place area (OPA) and retrosplenial cortex (RSC) were identified as the set of scene-selective voxels in the collateral sulcus, transverse occipital sulcus and medial parietal cortex (Epstein and Kanwisher, 1998; Nasr et al., 2011), the fusiform face area (FFA) was identified as the set of contiguous voxels showing face selectivity in the fusiform gyrus (Kanwisher et al., 1997), hMT+ was identified as the set of motion selective voxels in middle temporal cortex (Huk et al., 2002; Vangeneugden et al., 2014) and object-selective lateral occipital cortex (LO) was identified as the voxels situated posterior to hMT+ in the dorsal-caudal subdivision of lateral occipital complex (Grill-Spector et al., 2001). These regions of interest, similar to the master ROIs described above, were then used to identify participant-specific functional ROIs after being transformed to participants’ T1 space and intersected with individuals’ own thresholded activation maps.

Pericalcarine and basal ganglia ROIs [Nucleus Accumbens (NAc), Caudate, Putamen, Pallidum] were defined anatomically based on Freesurfers “aparc” automatic segmentation and orbitofrontal cortex (OFC) ROIs [lateral OFC (*l*OFC), medial OFC (*m*OFC), mid anterior OFC (*ma*OFC)] were identified with the OFC atlas (Öngür et al., 2003) included in FreeSurfer’s library. All ROIs were individually identified (laterally) and then combined to form bilateral ROIs. Average time series from each bilateral ROI were extracted using Nilearn’s NiftiLabelsMasker (Abraham et al., 2014). At the end of this procedure, 19 ROIs were created for most participants with the exception of four participants missing IPL and one participant missing vMPFC.

#### Activation Analysis of *a priori* ROIs

General linear model activation analyses were implemented in Python using Nistats (Abraham et al., 2014) and nltools (Chang et al., 2018) to characterize univariate activation in *a priori* ROIs in response to landscape movies (vs. baseline). Average timeseries data from voxels in each ROI were high pass filtered (0.01 Hz), detrended and *z* scored. The data across four runs were concatenated, design matrices were created by entering task block regressors for 30 s movie-on periods (separately for each four aesthetic appeal levels) and for overall rating response periods (4 s). The regressors were then convolved with a canonical hemodynamic response function (HRF). Motion parameters representing 3 translation and 3 rotation time-courses, their temporal derivatives, and quadratic terms of both were also included in the design matrices. From the resulting regression weights, linear contrasts for all movies vs. resting baseline were computed for each ROI and then compared to zero baseline at the group level using one-sample *t*-tests and corrected for multiple comparisons using Bonferroni correction (0.05/number of ROIs). The significance of the aesthetic appeal effect was tested using linear mixed effects analysis (with lmer function from lme4; Bates et al., 2015, implemented in R version 3.4.3) by adding linear contrasts for 4 vs. 1 and 4 vs. 321 aesthetic appeal and including intercepts for participants as random effects.

#### Whole Brain fMRI Analysis

Whole-brain fMRI data were analyzed with standard general linear models using tools from the FSL library [^3^ v5.0.11, FEAT, v6.00 (Smith et al., 2004) and Freesurfer]. Data were smoothed using a 5 mm Gaussian filter and high pass filtered (at 90 s). In the first level, a general linear model (GLM) analysis was implemented to extract separate parameter estimates for each of the 31 movie trials within every voxel in T1 space. Six motion parameters (three rotation and three translation), aCompCor (Behzadi et al., 2007) and FD (Power et al., 2014) values were added as nuisance regressors in this GLM. To identify regions showing sensitivity to overall aesthetic ratings of videos a second level fixed-effects analysis was computed with the responses of each participant on each of the four possible aesthetic appeal levels coded as separate regressors. Average motion energy values were calculated for each video and added as nuisance regressors. To identify regions sensitive to aesthetic appeal (4 vs. 1) and to further isolate processes particular to strong aesthetic responses (4 vs. 321) we computed two contrasts of interest for each participant. These contrast maps were then transformed to the Freesurfer group surface *fsaverage* (mri_vol2surf) after additional smoothing using a 5 mm FWHM kernel and a one-sample group mean statistical test was applied (mri_glmfit). The results were corrected for multiple comparisons using cluster-wise thresholding derived from Monte-Carlo simulations (mri_glmfit-sim) with a voxel-wise threshold of *p* < 0.001 and a cluster threshold of 0.05. We ran Freesurfer’s volumetric pipeline separately for the subcortical regions with the same parameters. Statistical corrections were done using three spaces (right, left hemisphere and subcortical).

#### Functional Connectivity Analyses

To obtain timeseries to use in the functional connectivity (FC) analysis, non-smoothed functional images were denoised using Nilearn (Abraham et al., 2014). We implemented voxel-wise confound regression by regressing out signals from six aCompCor components, 24 motion parameters representing 3 translation and 3 rotation time-courses, their temporal derivatives, and quadratic terms of both, outlier frames with FD > 0.5 mm and DVARS (Power et al., 2012) with a threshold of ±3 SD, together with their temporal derivatives, task effects and their temporal derivatives (Whitfield-Gabrieli and Nieto-Castañón, 2012), and any general linear trend. Time series were filtered using a 0.008–0.2 Hz band-pass filter.

First we extracted mean time series for each of the 19 ROIs (Figure 2A) separately for each run using Nilearn (Abraham et al., 2014). Then, we picked time points corresponding to each movie trial by controlling for delays due to the hemodynamic response function (HRF) (Whitfield-Gabrieli and Nieto-Castañón, 2012). In this procedure, we first convolved task block regressors with the HRF and applied a filter to retain only positive values of the resultant time series. This filter was applied to the original time series to retain 15 time points for each movie trial. Then, we calculated Pearson’s correlation coefficients between the mean signal time-course of node *i* and the mean signal time-course of node *j*, for all pairs of ROIs, for each movie trial. Finally, Fisher’s transformation was employed to convert Pearson’s correlation coefficients to normally distributed *z*-scores. This procedure resulted in 31 × 19 × 19 correlation matrices for each participant where the first dimension represents the number of movie trials and the last two dimensions a symmetric matrix containing 171 unique pairwise correlation values (edges). For each participant and edge, we computed linear regressions predicting the FC estimates with linear and quadratic regressors to explore connectivity patterns that showed modulations with aesthetic appeal. As a result of these regression analyses for both contrasts, we obtained *t*-values for every participant and edge. To identify edges with connectivity modulations that were different than zero we computed one sample *t*-tests for every edge with the values from each participant and computed significance, correcting for multiple comparisons using FDR.

##### Analysis with continuous ratings

To identify changes in FC related to specific moments of change in observers’ continuous ratings, we used a technique called multiplication of temporal derivatives (MTD) with a window length of 3 (7 total time points; Shine et al., 2015). We performed GLM analyses to model the MTD connectivity estimates using regressors created from the continuous ratings that indicated moments of change toward increasing or decreasing aesthetic enjoyment. The MTD timeseries and the regressors were concatenated across runs. The regressors were convolved with an HRF and downsampled to 0.5 Hz to match the time resolution of the functional data. Only three key nodes were included in this analysis: PPA, aMPFC and PCC. For the three unique comparisons that were possible across these three edges we compared the *t*-values for each regressor obtained from the GLM analyses for each participant with paired *t*-tests.

## Results

### Idiosyncratic Patterns of Aesthetic Enjoyment

Participants viewed movie clips of natural landscapes while rating their subjective level of aesthetic enjoyment at each moment (continuous rating), followed by a discrete judgment indicating the intensity of their aesthetic experience (overall rating; Figure 1A).

Despite mostly favorable overall ratings (Figure 1B), observers expressed a high degree of individuality, both in their distributions of responses and also in which clips they found most aesthetically appealing. Some observers tended to use only a small range of the scale, while others spread ratings across the full range (Figure 1C). The amount of “shared taste,” quantified by computing a “mean-minus-one” (MM1) correlation between each participant and group-averaged ratings (see *Methods*) was MM1 = 0.40 (95% CI 0.31–0.48; Figure 1D left). This is lower than the degree of agreement previously reported for still images of landscapes (MM1 = 0.60, 95% CI 0.53–0.66, Vessel et al., 2018). Partitioning the repeatable variance into shared and individual components (Germine et al., 2015; Martinez et al., 2020, see Materials and Methods) revealed that 12% of the variance in overall ratings was shared across participants, whereas 88% was attributable to individual taste.

To explore how subjective aesthetic enjoyment changed over time, we inspected the trial-by-trial continuous rating time series and again found large differences in individual responses, both across movies but also across participants. A measure of moment-to-moment agreement across participants (mean-minus-one “continuous” correlation, MM1c, see Materials and Methods) was MM1c = 0.61 (95% CI 0.52–0.69; Figure 1D, right) suggesting that moment-to-moment ratings were in fact more stable across people than overall ratings. Figure 1E depicts the continuous ratings from each participant for one movie (selected for showing the median MM1c score). Despite the higher average agreement on this measure, it is still clear that participants often showed divergent continuous rating profiles for the same movie, ranging from strongly liked to disliked, and from mostly flat to dynamically fluctuating.

A measure of motion energy computed from each video (see Materials and Methods) revealed that the amount of motion was not a significant driver of continuous nor overall aesthetic ratings: the mean correlation between participants’ continuous ratings and framewise motion energy was *r* = 0.03 (95% CI 0.01–0.04), and the mean correlation between average motion energy across each entire clip and overall ratings was *r* = 0.15 (95% CI 0.08–0.22).

### Landscape Movies Engaged Early Visual as Well as Ventral and Lateral Occipitotemporal Cortex

We characterized the strength of fMRI BOLD response to landscape movies in visual, reward and default-mode (DMN) brain regions using a set of independently defined regions-of-interest (ROIs; see *Methods*; Figure 2B). As expected, landscape movies strongly engaged all visual regions tested (Pericalcarine, FFA, LO, hMT+, OPA, PPA, RSC, *t*-tests vs. resting baseline, all *p’s* < 0.003, Bonferroni corrected). Highest average responses were observed in pericalcarine, LO, OPA and PPA. On the contrary, DMN regions were not, on average, consistently modulated by the landscape movies. Within reward regions, the lateral OFC was significantly activated by the landscape movies when compared to a resting baseline (one sample *t*-test, *t* = 5.49, *p* < 0.003, Bonferroni corrected), whereas the nucleus accumbens showed less activation compared to a resting baseline (one sample *t*-test, *t* = −5.34, *p* < 0.003, Bonferroni corrected).

Despite showing responsivity to the landscape movies, none of the *a priori* visual ROIs were significantly modulated by rated aesthetic appeal (Figure 2C), nor were reward or DMN ROIs (not shown). To overcome differences in scale use across observers, overall ratings from each participant were used to split the movies into four levels of aesthetic appeal. Contrasts of highly appealing vs. low appealing trials (“4 vs. 1”; Supplementary Table S1) and highly appealing vs. all other trials (“4 vs. 321”; Supplementary Table S2) did not reveal any significant modulations after correcting for multiple comparisons. At a less stringent, uncorrected threshold of *p* < 0.01, only OPA showed greater activity for highly appealing movies vs. other trials (4 vs. 321 contrast, *p* = 0.007).

### Aesthetically Appealing Movies of Natural Landscapes Engage Portions of Occipitotemporal Cortex and the Basal Ganglia

**FIGURE 3:**
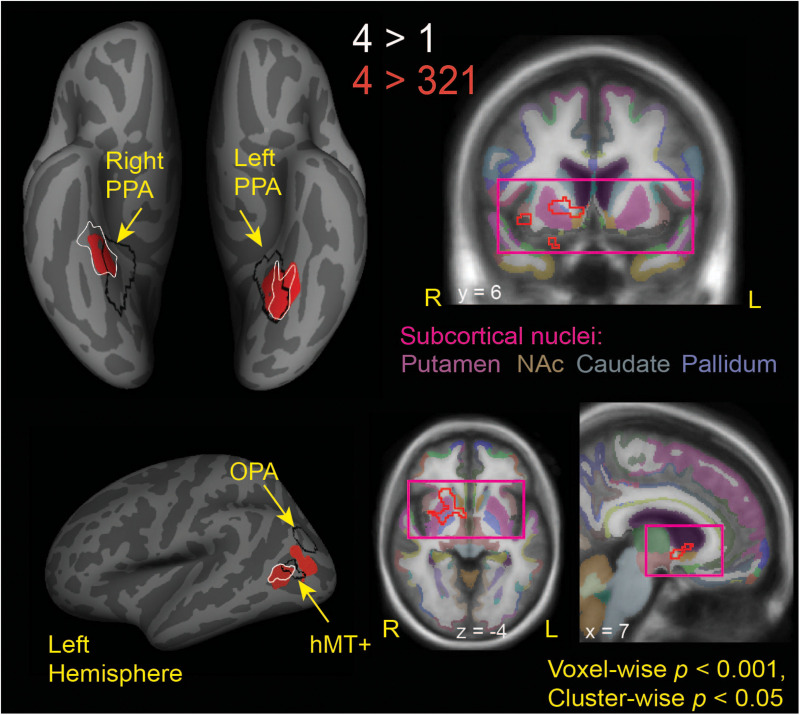
Brain regions modulated by aesthetic appreciation of landscape movies. Significant clusters of activations from a whole-brain beta series GLM analysis are shown for group contrasts of 4 vs. 1 (white outlines) and 4 vs. 321 (solid red: cortical, red outline: subcortical) levels of aesthetic appeal. *N* = 24. Black outlines show the scene-selective regions as found by the functional localizer task. PPA, parahippocampal place area; OPA, occipital place area; NAc, Nucleus Accumbens.

Looking beyond the *a priori* ROIs, a whole-brain analysis revealed several posterior cortical regions whose activity was modulated by aesthetic appeal. A contrast of high- vs. low- appeal movies (4 vs. 1) revealed activity in right and left collateral sulcus (CoS) and left posterior middle temporal gyrus (pMTG; Figure 3 white outlines, Table 1). A contrast of high-rated trials vs. all other trials (4 vs. 321) produced activations that overlapped with the 4 vs. 1 contrast in the CoS of both hemispheres and in the left posterior MTG, as well as an additional activation cluster in the middle occipital sulcus (MOS; Figure 3 solid red, Table 1).

**TABLE 1:** MNI coordinates for activations found in a whole-brain beta series GLM analysis for 4 vs. 1 and 4 vs. 321 contrasts.

Contrast	Hemisphere	MNI			Size mm^2^	Cluster-wise *p*	Max *p*	Location/BA
		*X*	*Y*	*Z*				
4 vs. 1	Left	−31	−64	−7	337	0.0036	1.54E-05	CoS, BA19-37
	Left	−46	−64	8	208	0.0414	2.06E-05	pMTG, BA19
	Right	37	−30	−23	651	0.0003	3.48E-07	CoS/PhG, BA37-36
4 vs. 321	Left	−32	−51	−12	610	0.0003	1.74E-05	CoS, BA19-37
	Left	−40	−70	20	369	0.0006	6.52E-05	MOS/MOG, BA18-19
	Left	−46	−63	7	307	0.0033	9.64E-06	pMTG, BA19
	Right	37	−42	−12	218	0.0220	5.48E-05	CoS/PhG, BA36
	
	**Subcortical**	***X***	***Y***	***Z***	**Size mm^3^**	**GRF Cluster-wise *p***	**Max *p***	**Location**
	
	Right	24	12	−4	5832	0.0006	1.55E-07	Anterior dorsomedial striatum (caudate and putamen), pallidum, NAc

*CoS, collateral sulcus; BA, Brodmann Area; pMTG, posterior middle temporal gyrus; PhG, parahippocampal gyrus; MOS, middle occipital sulcus; MOG, middle occipital gyrus; NAc, nucleus accumbens; GRF, Gaussian random field.*

Interestingly, the locations of these clusters sensitive to aesthetic appeal were adjacent to or even partly overlapping with regions of known stimulus selectivity from the localizer scans. The ventrally located clusters in CoS from both appeal contrasts partially overlapped with scene-selective cortex (PPA) as identified by the separate scene localizer scan, in both right and left hemispheres. In the right hemisphere, the effect of aesthetic appeal was shifted anterior and lateral from PPA, and in the left hemisphere it was shifted laterally. On the lateral surface, the more anterior appeal-sensitive cluster in pMTG was mostly anterior to the motion-sensitive hMT+, showing only a small degree of overlap, while the posterior cluster in MOS was situated between hMT+ and the scene-selective OPA (Figure 3).

The contrast of high-rated trials vs. all other trials (4 vs. 321) also produced a large subcortical cluster spanning several nuclei of the basal ganglia, including dorsal striatum (head of caudate [10, 12, 2], anterior putamen [24, 0, −2]) and anterior pallidum [20, 0, 0]. A small portion of this cluster also extended into ventral striatum [10, 7, −6] and anterior insula [39, 5, −6].

### Topographical Relationship Between Effects of Stimulus Content vs. Aesthetic Appeal for both Movies and Images of Natural Landscapes

**FIGURE 4:**
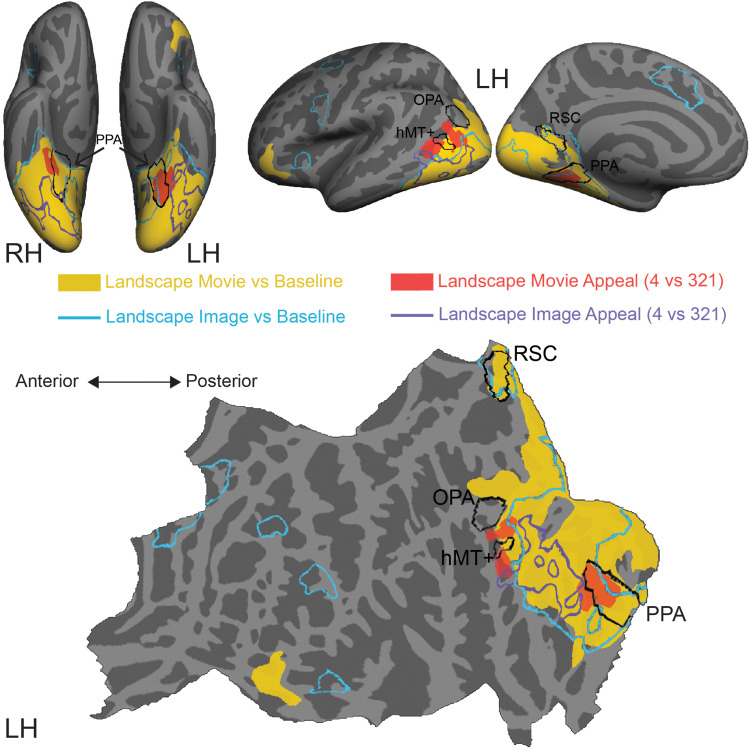
Comparison of stimulus-induced and appeal-related activations for both movies and still images of natural landscapes. Significant clusters from stimulus vs. baseline and aesthetic appeal contrasts (4 vs. 321) from two experiments were rendered on Freesurfer’s fsaverage cortical surface. Solid warm colors illustrate movie vs. baseline contrast (yellow) and effect of high appeal movies (4 vs. 321, red). Outlined cold colors illustrate still images vs. baseline contrast (teal) and effect of high appeal images (4 vs. 321, purple) obtained with static natural landscape images from Vessel et al. (2019). Top: inflated surfaces showing a ventral view of right (RH) and left (LH) hemispheres, and lateral and medial views of the left hemisphere. Bottom: flattened left hemisphere. Movie *N* = 24, Image *N* = 16.

We sought to more closely examine the topographical relationship between modulations of cortical activity by stimulus content vs. by aesthetic appeal. Using data from this experiment, recorded as observers judged the aesthetic appeal of movies of natural landscapes, as well as data from a previously published experiment in which a different set of observers made aesthetic judgments about still images of natural landscapes (Vessel et al., 2019), we compared content-related activations for both still images and movies with observed modulations by highly appealing stimuli (Figure 4).

In comparison to still images of landscapes (landscape images vs. resting baseline, blue outline), movies activated a greater extent of cortex (landscape movies vs. resting baseline, solid yellow, Supplementary Table S3), particularly dorsally, on both the lateral and medial surfaces. Activations for movies covered more of the lateral occipital cortex and extended into inferior and superior parietal regions. Additionally, movies of landscapes activated a region of inferior frontal gyrus and lateral orbitofrontal cortex on the left hemisphere and three more clusters around inferior frontal sulcus, inferior frontal gyrus and superior frontal sulcus on the right hemisphere. On the other hand, still images of landscapes produced activations in the isthmus of the posterior cingulate gyrus (retrosplenial cortex), as well as several prefrontal locations, including middle frontal and precentral gyri and small portions of lateral orbitofrontal gyrus and insula.

Turning to the modulations by aesthetic appeal, we found that the clusters on the lateral surface that showed greater activity for aesthetically appealing movies of natural landscapes (pMTG and MOS, Figure 4, filled red) sit at the anterior edge of the movie vs. baseline activation. This was not the case for the ventrally situated clusters in CoS. In addition, none of these clusters strongly overlapped the effect of aesthetic appeal observed for still images of natural landscapes, which extended across parts of the fusiform gyrus, lateral occipital cortex and inferior and middle temporal gyri (Figure 4, purple outline; data from Vessel et al., 2019).

### Changes in Functional Connectivity Associated with Aesthetic Appeal

We tested the hypothesis that aesthetic appeal is associated with changes in functional connectivity between content-sensitive visual regions and reward and DMN networks. To do so, we computed functional connectivity (FC) scores between each pair of *a priori* ROIs from the three networks, separately for each movie stimulus, using Pearson correlation (see Materials and Methods). We then modeled the trialwise FC estimates as a function of each observers’ overall aesthetic ratings using both linear and quadratic regressors, separately for each observer, and tested the resulting *t*-scores from these regressions for significance at the group level using one-sample *t*-tests (corrected for multiple comparisons using false discovery rate, FDR with *q* < 0.05; Benjamini and Hochberg, 1995).

**FIGURE 5:**
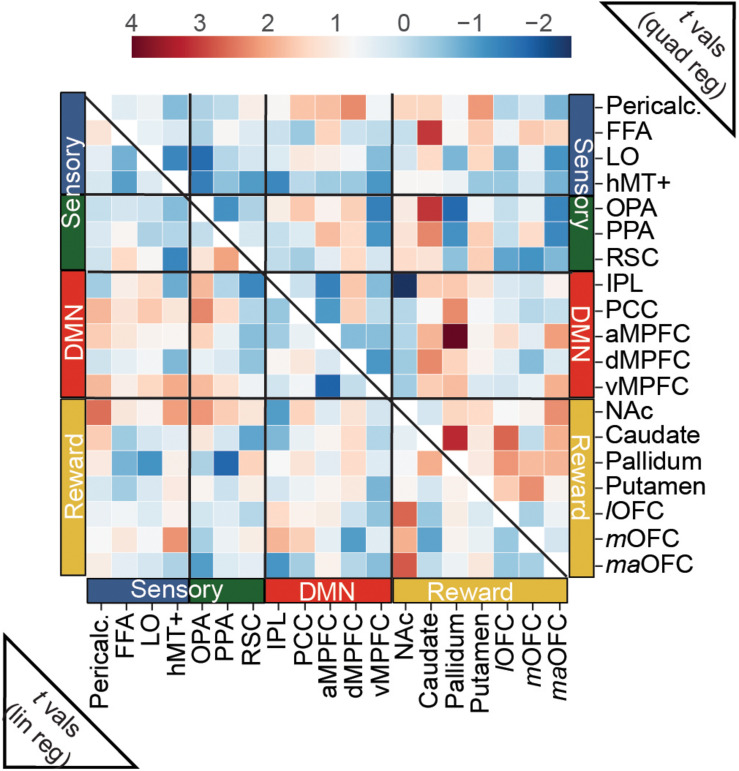
Predicting trial-wise functional connectivity estimates between *a priori* ROIs from visual, reward and DMN networks. FC scores were computed between each pair of *a priori* ROIs, separately for each movie stimulus, and modeled as a function of overall aesthetic ratings using both linear and quadratic regressors. Heat maps show *t*-scores from group level one-sample *t*-tests, conducted with the *t*-scores from the regressions for each edge. For the linear effect of aesthetic appeal (lower half), FC modulations were observed mainly between nucleus accumbens (NAc) and sensory ROIs and NAc and OFC ROIs (see text). For the quadratic effect (upper half) FC modulations were found between reward and sensory ROIs, between reward and DMN ROIs and within reward ROIs. *N* = 24. No scores were significant at *q* < 0.05 corrected for multiple-comparisons (false-discovery rate). One edge, (pallidum:aMPFC quadratic) was less than *p* < 0.001 (uncorrected).

Connectivity between our *a priori* ROIs was only weakly related to overall aesthetic appeal, as none of the edges tested in this analysis surpassed this stringent multiple comparisons threshold. However, when we examine the (uncorrected) pattern of average connectivity, several noteworthy relationships do emerge. For the linear effect of aesthetic appeal (Figure 5, lower half), connectivity between NAc and several visual regions increased with increasing aesthetic appeal [NAc:pericalcarine *t*(23) = 2.60, NAc:hMT+ *t*(23) = 2.14, NAc:OPA *t*(23) = 2.21], and NAc also showed increasing connectivity with several orbitofrontal regions [NAc:lOFC *t*(23) = 2.67, NAc:*ma*OFC *t*(23) = 2.76]. PCC, a core DMN region, showed stronger FC with OPA with increasing aesthetic appeal [PCC:OPA, *t*(23) = 2.36]. Increased connectivity was also observed between two nodes of scene-selective cortex, PPA:RSC *t*(23) = 2.08, and between *m*OFC:hMT+, *t*(23) = 2.24. The quadratic effect of aesthetic appeal was included to identify edges with highest FC for extreme values of aesthetic appeal regardless of direction (U-shaped relationship; Figure 5, upper half). Connectivity between the caudate and several sensory regions was modulated by aesthetic appeal in a quadratic fashion [caudate:FFA *t*(23) = 3.10, caudate:OPA *t*(23) = 3.11, caudate:PPA *t*(23) = 2.35], as was FC between several DMN and reward regions [pallidum:aMPFC *t*(23) = 4.01, caudate:dMPFC *t*(23) = 2.34, pallidum:PCC *t*(23) = 2.30, aMPFC:*ma*OFC *t*(23) = 2.13], and within several nodes of the reward network [caudate:pallidum *t*(23) = 3.22, caudate:lOFC *t*(23) = 2.66, putamen:mOFC *t*(23) = 2.29, pallidum:lOFC *t*(23) = 2.20, NAc:maOFC *t*(23) = 2.29]. Pericalcarine also showed a U-shaped relationship with one DMN [Pericalc:dMPFC *t*(23) = 2.31] and one reward node [Pericalc:Putamen *t*(23) = 2.17]. Finally, the connection between IPL and NAc showed a negative quadratic relationship to aesthetic appeal [IPL:NAc *t*(19) = −2.45].

We performed an additional analysis to test for possible changes in connectivity linked to moment-to-moment changes in aesthetic appeal, using observers’ continuous ratings of enjoyment. Restricting our analysis to 3 key ROIs (PPA, aMPFC, PPC) we modeled dynamic (time series) estimates of FC computed using the multiplication of temporal derivatives method (MTD, see Materials and Methods) with regressors coding for either positive or negative moments of change. None of the edges reached significance.

## Discussion

A core component of visual aesthetic experiences is the transformation of information about the contents of perception to a representation of aesthetic appeal. Using functionally defined ROIs, a robust approach more often used in studies of high-level vision, we tested whether independently localized posterior regions of the brain that represent perceptual features (e.g., scene layout or motion) are themselves modulated by aesthetic appeal. Using movies of natural landscapes, we found strong engagement of scene-selective regions in ventral (PPA) and medial (RSC) occipitotemporal cortex, as well as in lateral portions of visual cortex including scene-selective OPA, object-selective LO and motion sensitive hMT+. However, activity in these feature-selective regions was not significantly modulated by rated aesthetic appeal. Instead, we found greater activity for highly appealing movies in regions that were adjacent to and only partially overlapping with the feature selective ROIs, both on the ventral (bilateral CoS) and lateral (left pMTG and MOS) cortical surfaces. A large cluster spanning several nuclei of the right basal ganglia also showed increased activity for highly appealing movies. These findings suggest that regions representing core visual features such as scene shape and motion are not directly modulated by aesthetic appeal. Rather, adjacent cortex may be involved in the computation of information that is more directly relevant for aesthetic valuation. Additionally, contrary to what has been observed for artworks, we did not find evidence for modulation of prefrontal or default-mode regions correlated with aesthetic appeal. Finally, we did not find strong evidence for modulation of functional connectivity between feature-selective visual regions and nodes of reward or default-mode networks.

In contrast to previous studies using static images of scenes (Yue et al., 2007) and natural landscapes (Vessel et al., 2019), we did not find direct modulation by aesthetic appeal in scene-selective PPA, identified using an independent localizer scan (contrast of scenes vs. objects). Rather, a whole brain analysis identified bilateral clusters in CoS immediately adjacent to and partially overlapping with PPA that were more activated by the most appealing natural landscape videos. Note that at the level of individual observers, appeal-related effects were not reliably found in PPA. The apparent overlap on the left hemisphere is due to differences in the precise location of ROIs in individual observers compared to the rendered location of PPA on the group-averaged surface. These findings are generally consistent with many studies that have previously reported modulations by aesthetic appeal in portions of the ventral visual pathway (the “what” pathway; Goodale and Milner, 1992) thought to support an analysis of object and scene identity, though the precise locations of these activations have not been systematically investigated, and appear to vary with stimulus category (see Vessel, 2020).

Highly appealing movies of natural landscapes also led to greater activation in two clusters on the lateral surface, pMTG and MOS. Whereas modulation of lateral visual regions by aesthetic appeal has been reported in MOG for indoor built environments (Vartanian et al., 2013b), in hMT+ for kinetic dot patterns (Zeki and Stutters, 2012) and in LO for natural landscapes (Pegors et al., 2015) this is the first report of modulation by aesthetic appeal of natural landscape stimuli dorsal to LO. Similar to what was found on the ventral surface, these activations did not fall directly within scene-selective OPA nor motion-selective hMT+, but were rather adjacent. Increased engagement for dorsolateral portions of visual cortex, extending toward the dorsal visual stream (the “where” or “how” pathway; Goodale and Milner, 1992; Kravitz et al., 2011a), is likely a natural consequence of introducing motion in the stimuli (e.g., Zhao et al., 2020). However, modulation of the dorsal visual stream by aesthetic appeal is not well documented or understood (though see Calvo-Merino et al., 2008; Calvo-Merino et al., 2010; Zardi et al., 2021) for studies linking the aesthetics of dance and activity in extrastriate body area (Downing et al., 2001).

### From Visual Content to Aesthetic Appeal

Given previous work supporting the direct modulation of feature- or category-selective regions by aesthetic appeal (scenes in PPA but also for attractive faces in FFA, e.g., Iaria et al., 2008; Chatterjee et al., 2009; Pegors et al., 2015), it has been proposed that these regions might be directly involved in computations of aesthetic value (Chatterjee et al., 2009). Yet the results presented here suggest some care is needed. One important caveat is that only a subset of previous studies on aesthetic appeal have employed independent functional localizers, making it difficult to evaluate whether appeal-related activations fall precisely within feature- or category-selective regions. Furthermore, it is difficult to assess the degree to which appeal-related effects have been dissociated from stimulus-related effects across the literature. Faces and landscapes, in particular, tend to generate high agreement across people in which images they find appealing (“shared taste”; Hönekopp, 2006; Vessel et al., 2018). Thus, it is possible that previously reported activations in category-selective regions did not reflect a local representation of aesthetic appeal, but rather residual imbalances in visual features that were associated with aesthetic appeal and also led to greater activation of category-selective regions. For example, facial averageness correlates positively with average attractiveness ratings (Rhodes, 2006) and is also known to modulate FFA (Said et al., 2010) (albeit in the opposite direction). On the other hand, appeal-related activations have also been reported in occipitotemporal regions for stimulus sets with almost no shared taste, such as visual artworks (Vessel et al., 2012). Given that the movies used in this experiment generated low levels of agreement across participants’ overall ratings (only 12% “shared” variance), and no relationship was observed between motion energy measures and rated aesthetic appeal, it is likely that the activations reported here reflect processes related to aesthetic appeal rather than low-level stimulus characteristics. This may be one reason why the activations reported here appear mostly adjacent to, rather than in, category-selective PPA, hMT+ and OPA. This leaves us with the question of what is actually driving the observed responses. There are at least two potential answers to this question.

One possibility is that these activations adjacent to category-selective regions encode higher-level features that, while still “visual,” are not well captured by typical localizer contrasts. In the case of the ventral visual pathway, this could include factors such as visual openness (the degree to which a scene provides a wide angle of view; Greene and Oliva, 2009) and concreteness (the degree to which an image depicts specific representational content; Chatterjee et al., 2010). Both of these factors have been shown to be positively correlated with aesthetic ratings (Franz et al., 2005; Biederman and Vessel, 2006; Vartanian et al., 2015; Iigaya et al., 2020) and to modulate neural activity in higher level visual regions (though for openness in the opposite direction) (Henderson et al., 2011; Iigaya et al., 2020). In the case of the dorsal visual pathway, this may include higher-order motion cues (such as that observed in clouds), responses to optic flow, object tracking, or the degree to which a landscape affords exploration. If this were the case, the activations reported here (and potentially those observed in other studies of visual appeal) might reflect the extraction of such higher-order, non-local visual properties rather than true sensitivity to aesthetic appeal.

An alternative interpretation is that these activations adjacent to the category-selective regions reflect a transformation from a purely feature-based representation to a more complex, processing-based representation that reflects how a stimulus relates to an observers’ expectations, associations and past experiences. Information-based theories of aesthetic appeal propose that experiences are most pleasurable when they have the capacity to be understood while still providing novel information to the observer (Biederman and Vessel, 2006; Schmidthuber, 2010; Schoeller and Perlovsky, 2016) and thus optimize information acquisition. In the music domain, several recent studies have provided support for this idea, finding that pleasure in melodic sequences is highest when uncertainty (predictability, entropy) and surprise are balanced (Cheung et al., 2019; Gold et al., 2019). The movies used in this study afforded many opportunities for such novel, richly interpretable experiences, particularly given their use of novel viewpoints (from drones, helicopters or hard-to-reach locations), changes in perspective (panning, flying) and temporal modifications (double speed, time-lapse). These movies allowed participants to have experiences with the natural environment that are outside the typical range of experience, but not so strange as to be uninterpretable. The higher activation that we observed in CoS, MOS, and pMTG for highly appealing movies may therefore reflect a process that is not linked to any specific set of visual features, but rather to the informational richness of the observers’ experiences, which in turn results in reward and pleasure. More broadly, the wide variety of appeal-related activations reported in the ventral visual pathway for many different categories of stimuli may reflect such interactions between bottom-up stimulus-driven activity and top-down sense-making.

These sense-making processes likely represent an elemental form of affect. While affective factors such as valence (pleasure, displeasure) and arousal may only be explicitly represented in other, more specialized structures (Salzman and Fusi, 2010; Berridge and Kringelbach, 2013) there is accumulating evidence suggesting that affect related schemas (e.g., value, emotion) are embedded in the human visual system (Kragel et al., 2019) and that affective predictions already emerge during the early stages of visual object recognition (Barrett and Bar, 2009). Such local operations, occurring in sensory hierarchies, may contribute to affective appraisals of novelty and coping potential (Roseman and Smith, 2001; Silvia, 2005).

In a related manner, contextual associations, semantic interpretations and imagery triggered by a stimulus can also influence aesthetic appeal (Palmer and Schloss, 2010; Vessel and Rubin, 2010; Gartus and Leder, 2014; Belfi et al., 2018; Belfi, 2019; Levitan et al., 2020). Contextual associations, crucial for making sense of a scene, have been linked to activation in the parahippocampal gyrus (Aminoff et al., 2007), potentially overlapping with PPA. Stimuli that activate a broad network of semantic and contextual associations evoke strong activity in later stages of the ventral visual pathway that mediate object and scene recognition (Bar and Aminoff, 2003; Aminoff et al., 2007), increasing the interpretability of an experience and its associated pleasure (Biederman and Vessel, 2006). Several recent studies have reported activations falling anterior to the scene-selective regions in response to recall of scene-related visual information (Silson et al., 2019; Bainbridge et al., 2020). The activation cluster we found in the right CoS, just anterior to the PPA, may reflect engagement of memory-based representations of scene imagery and associations, in interaction with the ongoing stimulus. A similarly local mechanism, tuned to the detection of broadly associative activity in later states of perceptual pathways, could link such activity to affect (Biederman and Vessel, 2006). More generally, the transition from stimulus-linked activity to affectively tinged representations may align with other proposed gradients in higher sensory and associative regions, reflecting increasingly complex representations along a posterior to anterior gradient from hMT+ to perisylvian fissure; this transition has been suggested to represent a form of abstraction (Kable et al., 2002; Chatterjee, 2010).

### Movies Versus Still Images

When comparing the areas associated with aesthetic appeal of movies vs. images of landscapes, we found greatly increased engagement of lateral and dorsal regions for appealing movies. For the field, this finding points to the need for visual neuroaesthetics to avoid a narrow focus on the ventral visual pathway. On the ventral surface, we found that while appeal-related activations for movies were in the CoS and PHG, appeal for static images appeared to more strongly engage parts of the fusiform gyrus and LO. One possibility for this difference is that the increase in ecological validity afforded by movies improved their capacity to invoke contextual associations compared to scenes. Alternatively, the presence of motion-induced depth cues in movies may have driven attentional focus to scene shape and layout. Static images, lacking such cues, may permit greater attention to flow to individual object representations, and thus engage object-selective cortex such as LO to a greater degree. If this were the case, future research might test the prediction that aesthetic judgments of images are more determined by object content, whereas judgments of movies may be more related to scene shape than to object content. A final explanation to consider for some of the observed differences is that certain activations reported for static images may reflect short-lived effects related to the onset of a stimulus that are not maintained over the course of a 30 s movie.

### Engagement of Subcortical Reward Circuitry During Aesthetic Experiences With Natural Landscapes

We found a large cluster of activation extending over several nuclei of the right basal ganglia that responded more strongly to highly appealing movies, compared to other movies. This cluster was primarily contained within dorsal striatum structures such as caudate nucleus and putamen and included only a small fraction of ventral striatum (NAc). There is some discrepancy in the neuroaesthetics literature as to which structures of the basal ganglia are more relevant for aesthetic appeal, given that studies have reported activations in different areas (see Vessel, 2020). One study from the music domain proposed distinct roles for dorsal and ventral striatum in aesthetically appealing experiences; they proposed that activation in the caudate (dorsal striatum) is related to the anticipation of peak moments of pleasure, whereas NAc activation is involved in the consummatory aspects of musically induced pleasure (Salimpoor et al., 2011). A related proposal from the decision-making literature posits that dorsal striatum performs an “actor” function of learning and habit formation (Maia, 2009) and reward expectation (Delgado et al., 2000, 2003) and ventral striatum performs a “critic” function, representing actual rewards and reward prediction error (Schultz et al., 1992; Setlow et al., 2003; Wan and Peoples, 2006). Although this is a compelling theory, it failed to find support in a recent study that showed an opposite pattern for moments of poetry-evoked chills (Wassiliwizky et al., 2017) and in work implicating the NAc in tracking uncertainty during music rather than peak pleasure (Cheung et al., 2019).

An alternative explanation for the differential roles of ventral and dorsal striatum during aesthetically rewarding moments relates to the time scale of the reward prediction, rather than anticipation or consumption *per se.* Studies investigating reward prediction at different time scales have found that a number of areas in the limbic loop, including the ventral striatum, are involved in the prediction of immediate rewards; dorsal striatum, a part of the motor loop, was found to be involved in future reward prediction (Tanaka et al., 2004). The activation of dorsal striatum in response to aesthetically appealing landscape movies (this study) and images (Yue et al., 2007; Ishizu and Zeki, 2014) might relate to the fact that many of the pleasing features of landscapes relate to *potential* reward such as the potential for habitat, for exploration, for resource availability, or for monitoring one’s surroundings – environmental conditions favorable to survival (Rostrup, 2014).

### Lack of Modulation in Prefrontal and DMN Structures by Aesthetic Appeal

A secondary aim of this study was to evaluate whether aesthetically appealing natural landscapes lead to greater activation in *m*PFC, OFC or in nodes of the DMN. Many studies have reported modulation by aesthetic appeal in portions of *m*PFC, particularly in or around the superior rostral sulcus (Vessel, 2020). The *m*PFC, particularly vMPFC, is strongly implicated in the representation of subjective value (Bartra et al., 2013), and portions of the *m*PFC are also part of the DMN (Raichle et al., 2001; Andrews-Hanna et al., 2010). This finding has been particularly robust for aesthetically appealing artworks (Kawabata and Zeki, 2004; Vartanian and Goel, 2004; Lacey et al., 2011; Vessel et al., 2012; Belfi et al., 2019). In the current study, we did not find significant modulation by aesthetic appeal in *m*PFC, nor in independently localized nodes of the DMN, using both whole-brain and ROI-based analyses. One previous study with images of natural landscapes found sensitivity to landscape appeal only when using a small-volume correction in an *a priori* defined vMPFC ROI (Pegors et al., 2015). On the other hand, more sensitive, pattern-based analyses have found more robust information about the appeal of natural landscape images in *m*PFC (Pegors et al., 2015) and several nodes of the DMN (Vessel et al., 2019). However, more direct evidence for overall activation of DMN, *m*PFC or *m*OFC by landscapes is sparse.

The discrepancy between activation-based and pattern-based analyses may point to the relevance of spatial scale. Aesthetic appeal for landscapes may be represented in *m*PFC and other DMN nodes at a spatial scale that is too fine to survive an activation-based analysis, which requires a degree of spatial smoothing and averaging across observers. However, this possibility does not address the discrepancies observed for different aesthetic domains: sparse evidence for *m*PFC activation by appealing landscapes, but more robust evidence for appealing visual artworks.

One potential avenue for explaining this discrepancy may come from a differential contribution of bottom-up vs. top-down processing to appealing experiences with landscapes vs. artworks. Aesthetic experiences with artworks, more so than those with landscapes, may engage explicit, effortful top-down mechanisms supporting sense-making, imagery and resolution of ambiguities (Muth et al., 2015) incorporation of art-specific knowledge (Belke et al., 2006; Leder and Nadal, 2014), evaluation of artistic intent (Bullot and Reber, 2013) and assessments of self-relevance (Vessel et al., 2013; Pelowski et al., 2017). On the other hand, aesthetic experiences with natural landscapes, while clearly still engaging predictive and sense-making processes, may depend to a greater degree on bottom-up perceptual analyses and comparison to well-formed categorical representations of the natural world (Balling and Falk, 1982; Kaplan and Kaplan, 1989) developed over a lifetime of experience. Several lines of evidence support this interpretation. An experiment with natural landscape movies that evoked the feeling of awe (Keltner and Haidt, 2003), an aesthetic emotion closely related to beauty and being moved (Schindler et al., 2017), found that such movies were associated with a reduced sense of self and also reduced activity in core nodes of the DMN (*m*PFC, PCC; VanElk et al., 2019). Behaviorally, observers tend to agree on which natural landscapes they find appealing (high shared taste), but show much more individual taste for aesthetic judgments of artwork and architecture (Vessel et al., 2018). This increased agreement is due to the highly consistent way in which different observers semantically interpret a real-world scene compared to abstract images or artworks (Vessel and Rubin, 2010; Schepman et al., 2015; Leder et al., 2016) which itself may be a consequence of the behavioral relevance of understanding the physical landscape for everyday living, compared to a relative lack of daily relevance for assessments of artwork. Aesthetic responses to landscapes, then, may engage a set of interpretation processes that, while still able to generate surprise, contextual associations and rich interpretability, are more automated, more stereotyped, and less top-down. Indeed, a recent wave of research on nature’s capacity to reduce stress and improve cognitive functioning (Berman et al., 2008; Bratman et al., 2015a, b) posits that the “restorative” benefits of interacting with nature are on account of the natural environment’s ability to capture attention in a relatively effortless manner, while providing a degree of “mild fascination” (Kaplan and Kaplan, 1989).

Interestingly, in this and a previous study (Isik and Vessel, 2019) we observed that across-observer agreement for overall assessments of aesthetic appeal for movies of natural landscapes was significantly lower than that previously reported for still images of natural landscapes (Vessel et al., 2018). However, across-observer agreement for *moment-to-moment* ratings of enjoyment was higher. These findings suggest that moment-to-moment enjoyment of landscape movies may be a more bottom-up process without much need for integration from top-down mechanisms, but that subsequent overall assessments of these movies, performed after watching each clip, may draw on more top-down processes than is typical for images of natural landscape.

### Weak Evidence of Changes in Functional Connectivity Associated with Aesthetic Appeal

Our third aim was to test whether category-selective visual regions are functionally connected with prefrontal (Lim et al., 2013) or reward (Salimpoor et al., 2013) regions in a manner that is modulated by aesthetic appeal. Although we did observe a degree of FC modulation correlated with aesthetic appeal, particularly in connection with NAc, the effects were weak and did not reach statistical significance. Furthermore, although studies have reported increases in DMN’s intra-functional coupling during experiences with preferred music (Wilkins et al., 2014), we found only weak modulations involving DMN nodes for aesthetic appeal of natural landscape videos.

However, due to several limitations, it may be too soon to completely rule out the presence of such FC modulations. For example, it is possible that the moment-to-moment responses to the clips might not have varied enough to drive detectable fluctuations in functional connectivity; this possibility derives some support from an inspection of the continuous ratings, which were relatively flat for some observers. Additionally, the overall ratings skewed heavily positive, potentially resulting in a relatively weak contrast between the psychological states associated with high-rated and low-rated clips. This may have affected our ability to detect associated changes in functional network modulations. Alternatively, it is also possible that reward and DMN regions integrated information over time in a manner that blurred out such content-related fluctuations in functional connectivity (Simony et al., 2016). A further potential limitation may be related to length of the clips. It is possible that a 30 s time window (15 timepoints) was too brief for detecting FC fluctuations against a background of other high-frequency noise fluctuations in the BOLD signal. Some studies suggest using longer stimulus durations and sliding window correlations over longer window lengths in order to resolve lower frequency fluctuations of interest (Sakoǧlu et al., 2010). Finally, it is possible that brain connectivity dynamics may be more susceptible to alteration or masking by the requirement to make simultaneous, continuous ratings. Although existing behavioral (Isik and Vessel, 2019) and fMRI (Hutcherson et al., 2005) evidence provides no strong support for the alteration of participants’ aesthetic experiences due to simultaneous continuous ratings, several studies have claimed that explicit evaluations have an effect on functional connectivity dynamics (for music listening; Bogert et al., 2016; Liu et al., 2017).

## Conclusion

Natural landscapes are rich sources of aesthetic appeal, and by virtue of their central role in research on visual perception, can also serve as an important bridge between the fields of scene perception and neuroaesthetics. We conclude that aesthetic appeal *per se* is likely not represented in well-characterized feature- and category-selective regions of the visual system. Rather, we propose that modulations by aesthetic appeal observed in the visual system reflect a transformation from a feature-based visual representation to a representation of elemental affect, computed through information-processing mechanisms that rely on the detection of deviations from an observer’s expectations and activation of richly interpretable semantic and contextual associations. While the exact nature of these transformations remains unclear, we believe that these findings bring a new perspective to the difficult problem of how the brain computes affectively marked appraisals of aesthetic appeal from representations of visual content.

Our findings also hint at a potentially important difference between different aesthetic domains. While interactions between bottom-up and top-down processes likely play a role in the appreciation of all aesthetic experiences, the balance of these interactions may differ from one domain to the next. The evidence suggests that aesthetically appealing experiences with natural landscapes, in contrast to those with visual artworks, engage top-down processes supported by prefrontal cortex to a lesser degree.

## Data Availability Statement

The raw data supporting the conclusion of this article will be made available by the authors, without undue reservation.

## Ethics Statement

The studies involving human participants were reviewed and approved by the Medical Ethics Committee of Goethe University Frankfurt. The patients/participants provided their written informed consent to participate in this study.

## Author Contributions

AI and EV designed the study, interpreted the results, and wrote the manuscript together. AI collected and analyzed the data. Both authors contributed to the article and approved the submitted version.

## Conflict of Interest

The authors declare that the research was conducted in the absence of any commercial or financial relationships that could be construed as a potential conflict of interest.

## Footnotes

1 https://github.com/nipy/heudiconv

2 https://fmriprep.org/en/1.1.8/api/index.html#phase-encoding-polarity-pepolar-techniques

3 https://fsl.fmrib.ox.ac.uk/fsl/fslwiki
