# Supplementary material for: From Visual Perception to Aesthetic Appeal: Brain Responses to Aesthetically Appealing Natural Landscape Movies
Source: Front Hum Neurosci. 2021 Jul 21;15:676032. doi: 10.3389/fnhum.2021.676032 (PMC8336692; doi:10.3389/fnhum.2021.676032)
Supplement: Supplementary file 1 [file Data_Sheet_1.pdf]

Supplementary Material for Isik AI and Vessel EA (2021) From Visual Perception to Aesthetic Appeal: Brain Responses to Aesthetically Appealing Natural Landscape Movies. Front. Hum. Neurosci. 15:676032. doi: 10.3389/fnhum.2021.676032

Table S1 | Linear mixed model analysis results for the 4 vs. 1 contrast for each ROI with the beta values obtained from the ROI-based GLM activation analysis

**ROI name**	**Estimate**	***t***	***p***
pericalcarine	0.039	1.183	0.241
FFA	0.042	1.157	0.251
LO	0.080	2.582	0.012
hMT+	0.101	2.435	0.017
OPA	0.102	2.791	0.007
PPA	0.066	2.020	0.047
RSC	0.075	1.906	0.061
IPL	0.007	0.161	0.873
PCC	0.017	0.447	0.656
aMPFC	-0.034	-0.919	0.361
dMPFC	-0.011	-0.317	0.753
vMPFC	-0.013	-0.397	0.692
NAc	-0.013	-0.477	0.635
caudate	0.009	0.325	0.746
pallidum	0.025	0.915	0.363
putamen	-0.018	-0.575	0.567
*l*OFC	-0.028	-0.965	0.338
*m*OFC	0.033	1.174	0.245
*ma*OFC	0.000	0.006	0.995

Table S2 | Linear mixed model analysis results for the 4 vs. 321 contrast for each ROI with the beta values obtained from the ROI-based GLM activation analysis

**ROI name**	**Estimate**	***t***	***p***
pericalcarine	0.039	1.183	0.241
FFA	0.042	1.157	0.251
LO	0.080	2.582	0.012
hMT+	0.101	2.435	0.017
OPA	0.102	2.791	0.007
PPA	0.066	2.020	0.047
RSC	0.075	1.906	0.061
IPL	0.007	0.161	0.873
PCC	0.017	0.447	0.656
aMPFC	-0.034	-0.919	0.361
dMPFC	-0.011	-0.317	0.753
vMPFC	-0.013	-0.397	0.692
NAc	-0.013	-0.477	0.635
caudate	0.009	0.325	0.746
pallidum	0.025	0.915	0.363
putamen	-0.018	-0.575	0.567
*l*OFC	-0.028	-0.965	0.338
*m*OFC	0.033	1.174	0.245
*ma*OFC	0.000	0.006	0.995

Table S3 | MNI coordinates for activations found in a whole-brain GLM analysis for landscape movie vs baseline contrast

**Hemisphere**	**MNI** *X*	*Y*	*Z*	**Size mm^2**	**Cluster-wise *p***	**Max p**	**Location / BA**
Left	-12	-93	6	12520	0.0002	4.84E-18	Occipital lobe, fusiform, parahippocampal and lingual gyri, Precuneus
Left	-38	41	-11	683	0.0002	3.35E-06	Inferior frontal gyrus pars triangularis and orbitalis, Lateral orbito frontal gyrus
Right	14	-97	12	13367	0.0002	1.49E-17	Occipital lobe, fusiform, parahippocampal and lingual gyri, Precuneus
Right	37	8	23	367	0.0002	4.88E-05	Inferior precentral sulcus, Inferior frontal gyrus pars opercularis
Right	38	31	14	276	0.0024	6.71E-05	Inferior frontal sulcus, Rostral middle frontal gyrus
Right	24	0	47	219	0.01435	1.11E-06	Superior frontal sulcus, Precentral sulcus
